# Suspension Culture Alters Insulin Secretion in Induced Human Umbilical Cord Matrix-Derived Mesenchymal Cells 

**DOI:** 10.22074/cellj.2016.3987

**Published:** 2016-04-04

**Authors:** Fatemeh Seyedi, Alireza Farsinejad, Seyed Amirmahdi Nematollahi-Mahani, Touba Eslaminejad, Seyed Noureddin Nematollahi-Mahani

**Affiliations:** 1 Department of Anatomy, Afzalipour School of Medicine, Kerman University of Medical Sciences, Kerman, Iran; 2 Stem Cell Research Lab, Afzalipour School of Medicine, Kerman University of Medical Sciences, Kerman, Iran; 3 Department of Laboratory Medicine and Center for Bioscience, Karolinska Institute, Huddinge, Sweden; 4 Pharmaceutics Research Center (PRC), Neuroscience Research Center, Institute of Neuropharmacology, Kerman University of Medical Sciences, Kerman, Iran; 5 Physiology Research Center, Institute of Neuropharmacology, Kerman University of Medical Sciences, Kerman, Iran; 6 Afzal Research Institute, Kerman, Iran

**Keywords:** Suspension Culture, Wharton Jelly Cells, Insulin Producing Cell

## Abstract

**Objective:**

Worldwide, diabetes mellitus (DM) is an ever-increasing metabolic disorder. A promising approach to the treatment of DM is the implantation of insulin producing cells (IPC) that have been derived from various stem cells. Culture conditions play a pivotal role in the quality and quantity of the differentiated cells. In this experimental study, we have applied various culture conditions to differentiate human umbilical cord matrix-derived mesenchymal cells (hUCMs) into IPCs and measured insulin production.

**Materials and Methods:**

In this experimental study, we exposed hUCMs cells to pancreatic medium and differentiated them into IPCs in monolayer and suspension cultures. Pancreatic medium consisted of serum-free Dulbecco’s modified eagle’s medium Nutrient mixture F12 (DMEM/F12) medium with 17.5 mM glucose supplemented by 10 mM nicotinamide, 10 nM exendin-4, 10 nM pentagastrin, 100 pM hepatocyte growth factor, and B-27 serum-free supplement. After differentiation, insulin content was analyzed by gene expression, immunocytochemistry (IHC) and the chemiluminesence immunoassay (CLIA).

**Results:**

Reverse transcription-polymerase chain reaction (RT-PCR) showed efficient expressions of *NKX2.2, PDX1* and *INSULIN* genes in both groups. IHC analysis showed higher expression of insulin protein in the hanging drop group, and CLIA revealed a significant higher insulin production in hanging drops compared with the monolayer group following the glucose challenge test.

**Conclusion:**

We showed by this novel, simple technique that the suspension culture played an important role in differentiation of hUCMs into IPC. This culture was more efficient than the conventional culture method commonly used in IPC differentiation and cultivation.

## Introduction

Diabetes mellitus (DM) is the most common metabolic disorder that affects more than 5% of the global population (1). Two types of DM have been described, type I (insulin dependent) which is caused by the lack of insulin production, and type II (non-insulin dependent), which is caused by resistance to insulin and the lack of insulin receptors (2). Insulin dependent diabetes is usually caused by autoimmune damage of the pancreatic islet (3). Daily injections of insulin is a common treatment for diabetes type I but continuous daily injections reduce quality of life in patients and may cause problems such as angiopathy (4). Islet transplantation has long been suggested for treatment of type I DM which prevents secondary complications of DM, but inadequacies remain such as the limitation of the number of donor islets and probable immune system rejection (5,6). A promising approach to the treatment of DM is implantation of insulin producing cells (IPCs) (7,8). Current studies have shown that different stem cells could successfully differentiate into IPCs *in vitro* to be used as a new method for the treatment of DM (9). Human umbilical cord matrix-derived mesenchymal cells (hUCM), as a valuable source of stem cells, can be differentiated into IPCs after induction by pancreatic differentiation materials (10). Several factors may affect differentiation of the stem cells such as extracellular matrix (ECM) proteins, cell-to-cell adhesion, cell-to-cell contact, cell shape, surrounding forces and soluble factors (11). Previous studies have emphasized the cell shape as a potent regulator of cell growth and function (12) in addition to the ECM as an important regulator of cell fate through alteration of the cell shape (13). Although various stem cells have successfully been differentiated into IPCs (14), insufficient insulin production by *in vitro* generated IPCs is a serious obstacle following transplantation of *in vitro* differentiated cells as treatment of DM in animal models and humans (15). Research aims to achieve more simple and potent systems for the culture and differentiation of stem cells into IPCs. We report a novel, efficient and uncomplicated method for the differentiation of hUCMs into IPCs that has a higher yield of insulin secretion as confirmed by the glucose challenge test. 

## Materials and Methods

All materials were purchased from Sigma Company (USA) unless otherwise stated. The Ethics Committee at Kerman University of Medical Sciences, Kerman, Iran approved this experimental study. 

### Human umbilical cord matrix-derived mesenchymal cells harvest and cultivation

In this experimental study, we used a previously described method to harvest and culture hUCM (16). Briefly, human umbilical cord samples were collected from full term infants delivered by cesarean section following written consent from their mothers. The samples were transferred in Hank’s balanced salt solution to the laboratory. Under sterile conditions, Wharton’s jelly was cut into approximately 1 mm^3^pieces and cultured in Dulbecco’s modified eagle’s medium Nutrient mixture F12 (DMEM/F12) medium supplemented with 10% fetal bovine serum (FBS), 100 IU/ml penicillin, 100 mg/ml streptomycin and 1 μg/ml amphotericin B in 3 cm culture plates. The medium was changed every three days and the cells were passaged when they reached 80% confluency. The adherent cells were trypsinized and used for the experiments or cryopreserved for further use as described elsewhere (17). 

### Osteogenic and adipogenic differentiation

Osteogenic and adipogenic differentiation were carried out according to the method described by Gao et al. (18). hUCMs (1×10^4^cell/cm^2^) were detached from the substratum and cultured on glass slides. For osteogenic differentiation, the cells were fed with osteogenic medium that consisted of low glucose DMEM (L-DMEM) supplemented with 10% FBS, 10 nM dexamethasone, 50 μmol/L ascorbic acid and 10 mM L β-glycerol phosphate for 21 days. The induced cells were stained by alizarin red and observed under an inverted microscope (Olympus, Japan). For adipogenic differentiation, the cells were treated for 14 days with an adipogenic differentiation kit as recommended by the manufacturer (Invitrogen, USA, A1007001). Differentiated cells were stained by oil red O and analyzed by a light microscope. As controls, hUCM cells were maintained in the complete medium without inducing agents. 

### Flow cytometry

Viable hUCMs (1×10^5^ cells) were fixed with 4% paraformaldehyde for 15 minutes, washed by washing buffer, incubated with 200 μl of 10% goat serum for 15 minutes, and washed again by washing buffer, after which mouse anti-human antibodies conjugated with phycoerythrin (PE) against CD34, CD45, CD73, and CD90 were added. The mixture was incubated for one hour at 4˚C (19). The samples were washed by washing buffer and assessed by flow cytometry (BD FACS Calibur, USA). For each antibody, at least 10000 events were recorded per sample and data were analyzed by WinMDI software (BD Biosciences, CA). 

### Culture conditions and induction of human umbilical cord matrix-derived mesenchymal cells for insulin producing cell differentiation 

**Fig.1:**
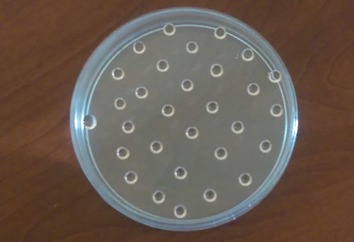
The cells were suspended in droplets of pancreatic induction medium and cultured for 14 days.

In the monolayer group, 4×10^5^viable hUCM cells were cultured in a 25 cm^2^culture flask. At 80% confluency the medium was changed to pancreatic medium. The cells were fed for 14 days and the medium refreshed every 3 days. In the hanging drop group, 1×10 ^4^hUCM cells were suspended in 50 µL droplets of pancreatic medium. The droplets were arranged on the lid of culture plates and the lid was carefully put on the top of the culture plates. Culture plates were filled with 5 ml sterile PBS to maintain sufficient humidity to protect droplets from dryness and osmolarity changes (Fig.1). Pancreatic medium consisted of serum-free DMEM/F12 medium with 17.5 mM glucose supplemented by 10 mM nicotinamide, 10 nM exendin-4, 10 nM pentagastrin, 100 pM hepatocyte growth factor, B-27 serum-free supplement (Gibco, Australia), 1% penicillin/streptomycin and 1 μg/ml amphotericin B. 

### RNA extraction and cDNA synthesis

Total RNA was extracted using a total RNA purification kit (Jena Bioscience, Germany) according to the manufacturer’s protocol. RNA samples were treated with DNase (Ambion, UK) in order to remove possible contaminating genomic DNA. The yield of RNA was evaluated by UV spectroscopy (NanoDrop Thermo, USA). We used 100 ng of total RNA for cDNA synthesis as described by the manufacturer (Bioneer AccuPower® Cycle Script RT Premix). 

### Reverse transcription-polymerase chain reaction

RT-PCR was performed by using a thermal cycler (Bio-Rad c1000, USA) and the following primer sequences: *GAPDH* (87 bp) F: 5´-TGCACCACCAACTGCTTAGCT-3´, R: 5´-GGCATGGACTGTGGTCATGAG-3´; NKX2.2 (228 bp) F: 5´-ATGTAAACGTTCTGACAACT-3´, R: 5´-TTCCATATTTGAGAAATGTTTGC-3´; INSULIN (192 bp) F: 5´-TTCTTCTACACACCCAAGAC-3´, R: 5´-CTAGTTGCAGTAGTTCTCCA-3´; and PDX1 (163 bp) F: 5´-CTGTGCTCCAGTTCCACACT-3´, R: 5´-ACAGCCTCTACCTCGGAACA-3´. 

The PCR reaction was performed on 20 µL of the following mixture: 10 µL master mix (Amplicon, Denmark), 1 µL sense primer (10 pm/L), 1 µL antisense primer (10 pm/L), 6 µL H_2_O, and 2 µL cDNA. PCR was carried out for 35 cycles: primary denaturation for 4 minutes at 94˚C, denaturation for 30 seconds at 94˚C, annealing for 30 seconds at 58-60˚C, and extension for 30 seconds at 72˚C, with an additional 5 minute incubation at 72˚C at the end of the cycle. The PCR products were visualized by running on 2% agarose gels. 

### Immunocytochemistry

Induced cells were fixed in 4% paraformaldehyde for 30 minutes, then washed three times by phosphate buffer saline (PBS), incubated with 0.5% Triton X-100 in PBS, incubated in 10% goat serum for 45 minutes at room temperature, followed by a final incubation overnight with guinea pig anti-insulin antibody (Abcam, USA) at 4˚C. 

The next day, the cells were washed three times with PBS and incubated with secondary antibody, goat anti-guinea pig fluorescein isothioc (FITC, Abcam, USA) for 45 minutes at room temperature. 

The nuclei were visualized by incubation of slides in 5 µg/mL Hoechst 33258 for 10 minutes after three PBS washes. The cells were analyzed by a fluorescence inverted microscope equipped with a digital camera (DP71, Olympus, Japan). 

### *in vitro* determination of insulin levels

The differentiated cells were washed twice with PBS and placed in L-DMEM that contained 0.5% BSA for 12 hours until all glucose within the cells were expended. The cells were then incubated in high glucose DMEM (H-DMEM, 25 mM glucose) and L-DMEM (3 mM glucose) for 2 hours. The supernatants obtained from low and high glucose cultures were collected, centrifuged and stored at −20˚C. Secreted insulin levels were determined by the human ultra-sensitive chemiluminesence immunoassay (CLIA) method (Liaison, DiaSorin). 

### Statistical analysis

The experimental data were analyzed using SPSS 21 for Windows (SPSS Inc., USA), employing basic statistical techniques. Other appropriate statistical software such as Microsoft Office Excel 2010 was also used. The independent samples t-test was applied to determine the difference between the treatment groups. A difference of P<0.05 was considered statistically significant. 

## Results

### Morphologic characteristics of human umbilical cord matrix-derived mesenchymal cells

**Fig.2:**
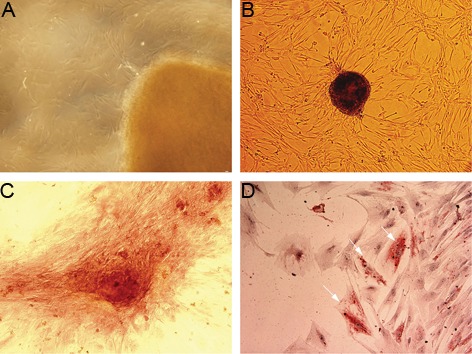
A. Human umbilical cord matrix-derived mesenchymal cells (hUCM) propagated from the boundary of Wharton’s jelly fragments, B. Colony formation in hUCM cells, C. Ca2+ aggregation in the extracellular matrix (ECM) was shown by alizarin red staining and D. Oil red O staining showed the presence of lipid droplets in the cytoplasm of the induced cells (magnification A, B: ×100, C and D: ×200).

At 7-10 days after the start of the explants culture, hUCM cells propagated from the boundary of the Wharton’s jelly fragments (Fig.2A). Propagated cells differed in size and showed both a spindle shape and round appearance with high mitotic activity (data not shown). After the first passage the fibroblast like cells with numerous cytoplasmic extensions propagated well in the culture and formed colonies (Fig.2B). The morphology of the cells remained rather unchanged within the passages. 

### Osteogenic and adipogenic differentiation

We cultured hUCM cells in osteogenic medium for 21 days. Next, the induced cells were examined for the presence of Ca^2+^ aggregates in the ECM. Alizarin red staining showed a positive reaction within the induced cells (Fig.2C). hUCM cells were also seeded in adipogenic medium for 14 days and lipid vacuolization was assessed by oil red O staining. Lipid droplets were detected in the induced cells (Fig.2D). Neither osteogenic nor adipogenic properties were observed in the control groups. 

### Flow cytometry analysis

**Fig.3:**
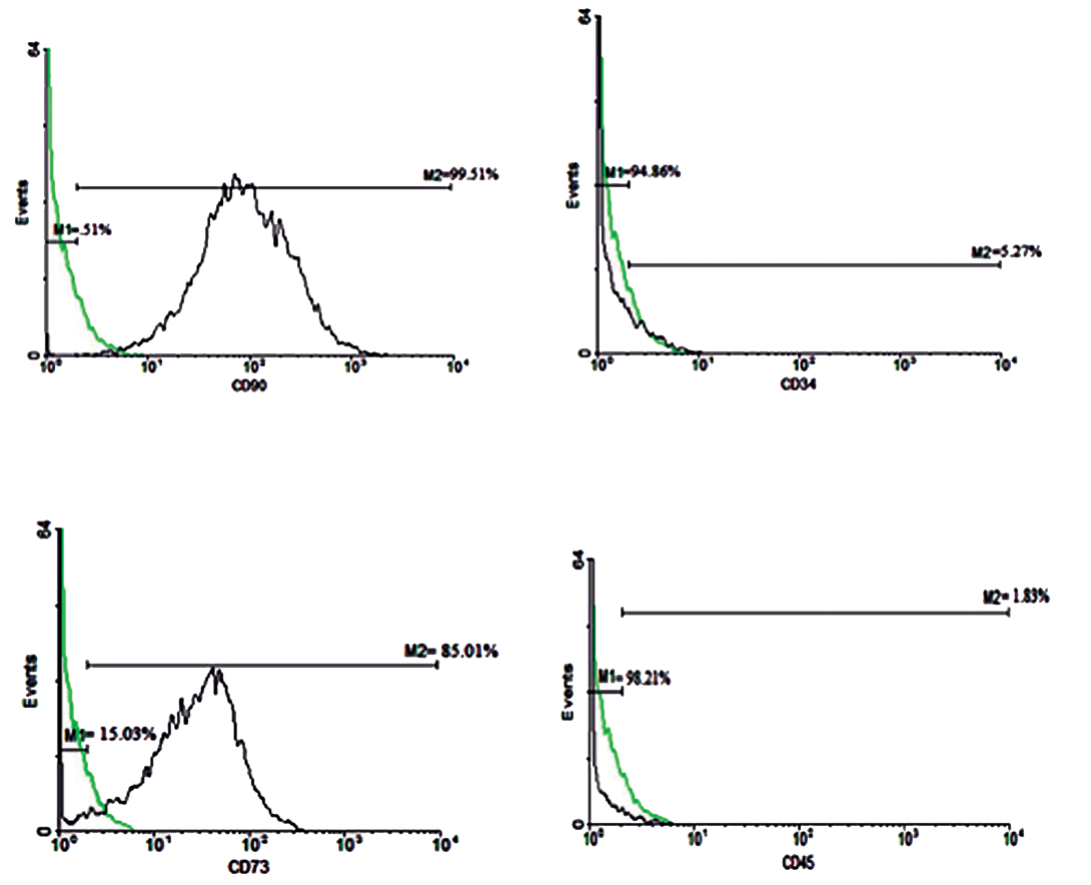
Flow cytometry analysis of mesenchymal stem cell (MSC) marker expression in human umbilical cord matrix-derived mesenchymal cells (hUCM) shows the expression of MSC markers CD90 and CD73, and lack of expression of CD34 and CD45. The respective immunoglobulin isotypes were used as negative controls. Green histograms show the isotype control-stained cells and black shows the antibodystained cells.

Flow cytometry analysis showed that hUCM cells expressed the mesenchymal stem cell (MSC) markers CD90 and CD73, but did not express CD34 (hematopoietic cell marker) and CD45 (leukocyte common antigen, Fig.3). 

### Insulin producing cell differentiation

**Fig.4:**
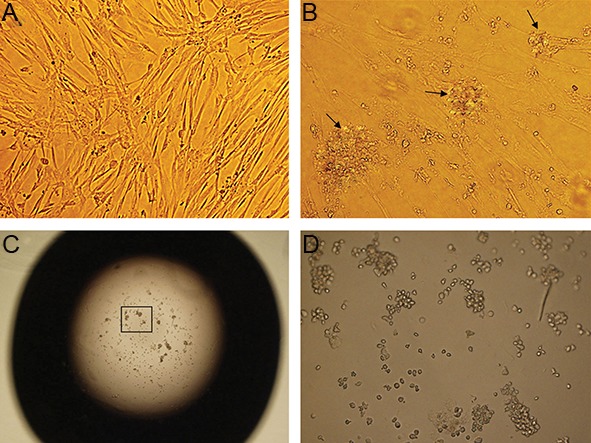
A. Morphology of passage 3 human umbilical cord matrix-derived mesenchymal cells (hUCM), B. Morphological changes following pancreatic differentiation in the monolayer, C. and D. hanging drop culture after 14 days (magnification A, B, D: ×200 and C: ×40)

In the monolayer group, undifferentiated hUC-Ms which were typically adherent spindle shape became round, aggregated and finally formed cell clusters under pancreatic induction conditions (Fig.4A,B). The cells in the hanging drops aggregated rapidly and formed cell clusters during the initial hours of culturing. Cell aggregates were detectable under an inverted microscope (Fig.4C,D). 

### Gene expression analysis

**Fig.5:**
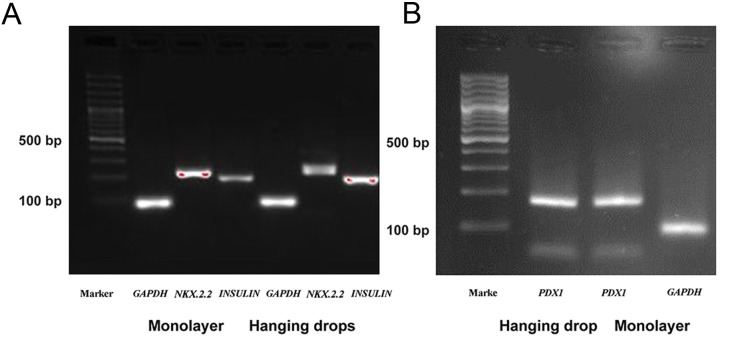
Gene expressions in the monolayer and hanging drops groups. A. *GAPDH* (87 bp), *NKX2.2* (228 bp) and *INSULIN* (192 bp) and B. Gene expressions in the monolayer and hanging drop groups expressed PDX1 (163 bp) with *GAPDH* as the reference gene.

In order to determine whether hUCM cells underwent differentiation into IPCs, we assessed gene expression by RT-PCR in both the hanging drop and monolayer cultures. *NKX2.2, PDX1* and *INSULIN* genes were expressed in both groups when compared with *GAPDH* as an internal control gene (Fig.5A,B). 

### Immunocytochemistry analysis

**Fig.6:**
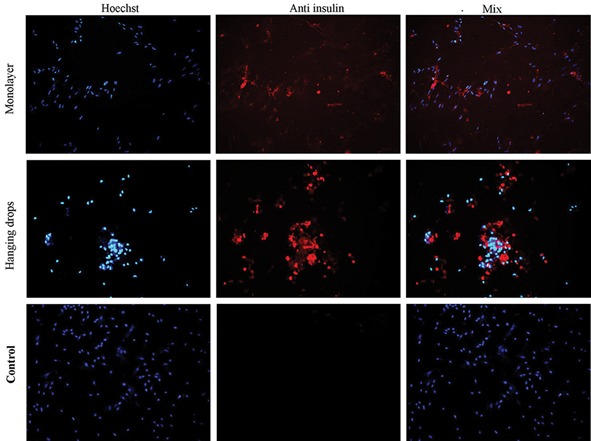
Immunocytochemical analyses were carried out on the hanging drops, monolayer and control groups for insulin protein expression after pancreatic differentiation for 14 days. The cell nuclei were counterstained with Hoechst (magnification: ×100).

Immunocytochemical analysis was performed on hUCM cells cultured in pancreatic differentiation medium for 14 days. Differentiated cells in hanging drops and the monolayer culture expressed the insulin protein. The proportion of insulin positive cells in the monolayer group was 45 ± 4.16, while in the hanging drops group it was 72.75 ± 4.19 (Fig.6). 

### *in vitro* determination of insulin levels

**Fig.7:**
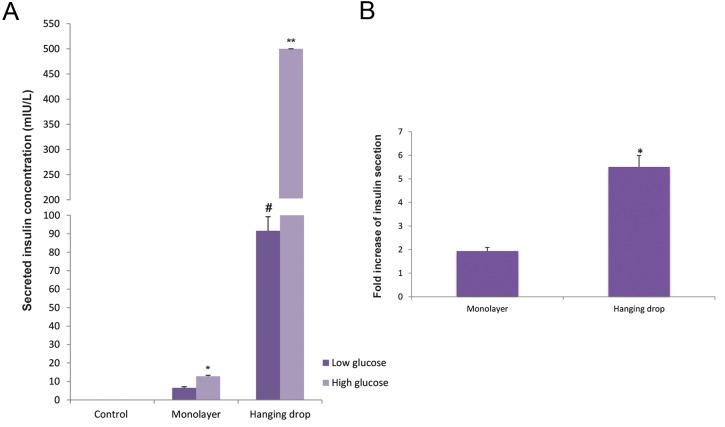
Insulin secretion was assayed in the monolayer, hanging drops and control groups following exposure to high and low glucose media. Insulin secretion following culture in low glucose medium was significantly higher in the hanging drops group compared with the control and monolayer groups (#; P<0.000). Insulin secretion in high glucose medium was significantly higher in the monolayer group compared with the control group (*; P<0.02), while in the hanging drop group insulin secretion was significantly higher when compared with the control and monolayer groups (**; P<0.000); (A. n=3-5). Fold increase of insulin secretion following glucose challenge test was also significantly higher (*; P<0.004) in the hanging drop group compared with the monolayer group (B. n=3-5). Data were expressed as mean ± SE.

The supernatants from the hanging drops and monolayer cultures were collected and the amount of insulin was measured by CLIA. The results indicated that insulin secretion during the exposure of IPCs to high glucose medium was higher than low glucose medium in both systems. Insulin secretion in the L-DMEM medium was 6.58 ± 0.57 mIU/L in the monolayer culture and 90 ± 7.6 mIU/L in hanging drops. In H-DMEM medium, this value increased to 12.86 ± 0.55 (monolayer) and ≥500 (mIU/L) for the hanging drops (Fig.7A). Glucose challenge test revealed a 2-fold increase in the monolayer culture and a 5.5-fold increase in the hanging drops (Fig.7B). 

## Discussion

According to the results, hUCM cells successfully differentiated into IPCs in two-dimensional (2D) and three-dimensional (3D) environments, as confirmed by gene expression and protein synthesis. CLIA evaluation of *in vitro* insulin secretion showed that both groups secreted insulin and responded to the glucose challenge test, however the rate of insulin secretion in the hanging drops group was significantly higher (about 40-fold) than the monolayer group. Exposure of induced cells to low and high glucose environments resulted in 2-fold insulin secretion in the monolayer group and a 5.5-fold insulin secretion in the hanging drops group. *In vitro* exposure of islets of Langerhans to low and high glucose conditions was reported to accelerate insulin secretion at a rate of 3-to 4-fold (20,21), higher than observed in the monolayer group but lower than the hanging drop group in our experiments. It could be concluded by comparing our results with the results reported thus far from the *in vitro* culture of islets of Langerhans that the hanging drop system might work similar to *in vitro* islet cells insulin secretion. 

Until now, a number of studies used different protocols to differentiate stem cells into IPCs. Wang et al. (5) reported insulin secretion of 9.54 ± 4.86 mmol/L in induced Wharton’s jelly cells without the glucose challenge test. Kim et al. (22) compared the IPC differentiation ability between four types of MSCs derived from bone marrow, adipose tissue, periosteum and Wharton’s jelly. Although gene expression and protein secretion was observed in all groups of cells, only IPCs derived from the periosteum efficiently responded to the glucose challenge test. In another study, Wu et al. (23) differentiated hUCMs and bone marrow–derived MSCs (BM-MSCs) into IPCs. They reported higher insulin secretion in hUCMs (35 mIU/L) following the glucose challenge test compared to BMMSCs (25 mIU/L). The current study used the glucose challenge test as a reliable physiologic test for the evaluation of insulin secretion. The results have shown a large amount of insulin secretion in the 3D culture (≥500 mIU/L) compared with 13 mIU/L in the 2D culture. The cell environment, including the cell niche and culture conditions play an essential role in cell fate and their behavior (24). Cell migration, proliferation, differentiation and apoptosis is affected by the ECM (25). The ECM is composed of soluble and insoluble factors that affect stem cell differentiation. The soluble factors consist of growth factors and cytokines, whereas insoluble factors comprise cell adhesion molecules, cell shape, mechanical forces from the surrounding environment, and substrate rigidity (11). A number of related studies have shown that the ECM affects cell fate through a change in the cell’s shape (13). The cell shape is a potent regulator of cell growth and function (12) as well as embryonic development and stem cell differentiation (13). For example, a change in the cell shape has been implicated as a potential mechanism that regulates myocardial development (26). Maintenance of chondrocyte shape in a round configuration has been shown to increase synthesis and secretion of sulfate matrix such that growth of the chondrocytes in a flat (2D) culture caused them to dedifferentiate and shift into a fibroblast phonotype. However maintenance of chondrocytes in the 3D environment preserved their normal phonotype (13). A round shape in lipid cells allows them to save maximum lipid droplet in adipose tissue, while cell spreading facilitates osteoblast matrix formation (27). Changes in morphology are created by changes in cadherin, integrin and other cytoskeletal protein expressions during stem cell commitment (28). McBeath et al. (27) have demonstrated that the cell shape regulated commitment of human MSCs to an adipocyte or osteoblast fate. The round shape of cells in hanging drops might have led to a higher degree of differentiation into IPCs. This was confirmed by IHC results where the number and intensity of the IPCs was prominent in the 3D culture and allowed the cells to secrete more insulin granules than the cells in the monolayer culture. 

Cell to cell adhesion is another important factor in the stem cell fate (13). An essential molecule in cell-cell adhesion is cadherin (29). Minami et al. (30) have shown that pancreatic exocrine acinar cells can change into pancreatic endocrine cells in the presence of cadherin molecules. Cadherin inhibition blocks trans-differentiation into IPCs. The use of an antibody against E-cadherin suppresses genes that induce pancreatic characteristics. In the hanging drops method, all of the cells aggregated in the center of the drops due to gravity and made a rather homogenous cell cluster. Cell shape, cell aggregation, and cell clustering in IPCs could have resulted in the higher insulin production in the 3D culture condition. 

## Conclusion

We have shown by a novel and simple method that differentiation of human umbilical cord matrix derived mesenchymal cells into insulin secreting cells is more efficient in 3D culture than 2D culture in protein continuance, insulin secretion, and response to the glucose challenge test. 
